# Combinatorial Conflicting Homozygosity (CCH) analysis enables the rapid identification of shared genomic regions in the presence of multiple phenocopies

**DOI:** 10.1186/s12864-015-1360-4

**Published:** 2015-03-10

**Authors:** Adam P Levine, Thomas M F Connor, D Deren Oygar, Guy H Neild, Anthony W Segal, Patrick H Maxwell, Daniel P Gale

**Affiliations:** Division of Medicine, University College London, London, UK; Nicosia State Hospital, Burhan Nalbantoğlu State Hospital, Nicosia, North Cyprus; School of Clinical Medicine, Cambridge University, Cambridge, UK; UCL Centre for Nephrology Rowland, Hill Street, Royal Free Hospital, Rowland Hill Street, London, NW3 2PF UK

**Keywords:** Linkage, Identical by descent, Phenocopy, Haplotype, Pedigree

## Abstract

**Background:**

The ability to identify regions of the genome inherited with a dominant trait in one or more families has become increasingly valuable with the wide availability of high throughput sequencing technology. While a number of methods exist for mapping of homozygous variants segregating with recessive traits in consanguineous families, dominant conditions are conventionally analysed by linkage analysis, which requires computationally demanding haplotype reconstruction from marker genotypes and, even using advanced parallel approximation implementations, can take substantial time, particularly for large pedigrees. In addition, linkage analysis lacks sensitivity in the presence of phenocopies (individuals sharing the trait but not the genetic variant responsible). Combinatorial Conflicting Homozygosity (CCH) analysis uses high density biallelic single nucleotide polymorphism (SNP) marker genotypes to identify genetic loci within which consecutive markers are not homozygous for different alleles. This allows inference of identical by descent (IBD) inheritance of a haplotype among a set or subsets of related or unrelated individuals.

**Results:**

A single genome-wide conflicting homozygosity analysis takes <3 seconds and parallelisation permits multiple combinations of subsets of individuals to be analysed quickly. Analysis of unrelated individuals demonstrated that in the absence of IBD inheritance, runs of no CH exceeding 4 cM are not observed. At this threshold, CCH is >97% sensitive and specific for IBD regions within a pedigree exceeding this length and was able to identify the locus responsible for a dominantly inherited kidney disease in a Turkish Cypriot family in which six out 17 affected individuals were phenocopies. It also revealed shared ancestry at the disease-linked locus among affected individuals from two different Cypriot populations.

**Conclusions:**

CCH does not require computationally demanding haplotype reconstruction and can detect regions of shared inheritance of a haplotype among subsets of related or unrelated individuals directly from SNP genotype data. In contrast to parametric linkage allowing for phenocopies, CCH directly provides the exact number and identity of individuals sharing each locus. CCH can also identify regions of shared ancestry among ostensibly unrelated individuals who share a trait. CCH is implemented in Python and is freely available (as source code) from http://sourceforge.net/projects/cchsnp/.

**Electronic supplementary material:**

The online version of this article (doi:10.1186/s12864-015-1360-4) contains supplementary material, which is available to authorized users.

## Background

Genetic analysis of multiply affected pedigrees has seen a resurgence of interest in the post-GWAS era [1,2]. Typically, in order to identify parts of the genome that segregate with a trait in a pedigree, linkage analysis is performed using haplotypes reconstructed from a large number of biallelic single nucleotide polymorphism (SNP) genotypes. Although the underlying SNP genotyping is robust and inexpensive, the analysis can be computationally demanding and time consuming. While effective and rapid methods exist for mapping homozygous alleles responsible for recessive traits in consanguineous families, identification of shared heterozygous variants causing dominant traits can be more computationally demanding. Owing to the exponential increase in computational requirements with increasing pedigree size for exact algorithms (e.g. Lander-Green), large families (especially those with many non-founders) pose a particular analytical challenge. Two commonly utilised approaches include pedigree splitting [3], which can result in significant loss of power [4], or the use of Markov chain Monte Carlo (MCMC) approximation [5]. The latter can still be computationally intensive, time consuming, and can fail to converge. Some of these problems have been overcome by a recent parallel implementation of the MCMC method [6].

A second problem in the analysis of extended pedigrees is that if some individuals are phenocopies (individuals that share the phenotype under consideration but not the genetic variant responsible for the trait in other family members) this causes erroneous exclusion of the causative locus, since phenocopies lack the true disease-linked haplotype. Although parametric linkage analyses can be performed under the hypothesis of a specified phenocopy rate, *a priori* this rate is unknown and analyses are sensitive to model misspecification [7] so analyses may have to be repeated across a range of parameters and significance thresholds adjusted accordingly [8]. Even utilising an appropriate phenocopy rate or conducting non-parametric linkage can result in failure to identify the responsible locus or prioritisation of the incorrect locus [9]. Furthermore, including phenocopies in whole genome or whole exome sequencing strategies will similarly prevent detection (or lead to erroneous exclusion) of the causative variant. These considerations have limited genetic studies in single families to those in which the phenotype is so rare or distinctive that phenocopies are extremely unlikely.

We have developed a tool called Combinatorial Conflicting Homozygosity (CCH) that addresses these challenges. Rather than calculating the likely flow of haplotypes through a family, CCH uses dense SNP genotypes to identify individual markers that cannot have been inherited identical-by-descent (IBD) among a set of individuals from a recent common ancestor. This allows identification of regions of the genome that contain consecutive markers in which IBD inheritance is not excluded. We show that above a threshold size (of 4 cM in the example here) the probability that such regions are *not* the result of IBD inheritance becomes vanishingly small. This *exclusion* approach contrasts with homozygosity mapping (which is used to map recessive, not dominant alleles) in which identical homozygous genotypes define the subset of the genome *included* in analysis. We demonstrate how CCH can be used to identify regions of the genome where a single haplotype is shared by some, but not all, members of a family and that this information can be used to identify individuals harboring a heterozygous disease-linked variant in a family with multiple phenocopies, and compare CCH with traditional parametric linkage analysis. Unlike pedigree-based linkage methods used to analyse dominant traits, this approach is equally applicable to related and unrelated individuals or combinations thereof.

## Methods

### CCH algorithm and implementation

**Figure 1:**
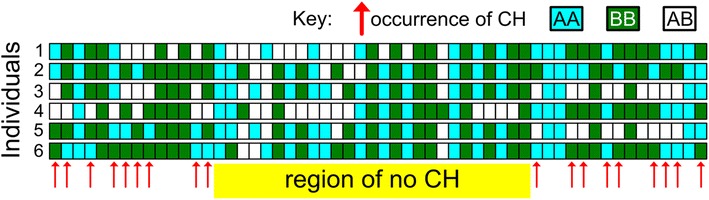
**Conflicting homozygosity (CH) occurs when two or more individuals are homozygous for different SNP alleles (e.g. AA and BB) and indicates that these individuals cannot all share a haplotype spanning this SNP.** The absence of CH is consistent with identical by descent inheritance at that locus.

The underlying algorithm of CCH is based on the simple, previously described principle that inheritance of at least one shared haplotype among two or more individuals is excluded by the occurrence of SNPs homozygous for different alleles, i.e. identical-by-state for zero alleles (IBS0) [10,11] (a situation we term conflicting homozygosity, CH, Figure 1). CCH uses this principle to infer IBD inheritance at loci where, in a group of individuals, CH is not observed across numerous consecutive SNPs. To do this, the number of consecutive SNPs for which CH is not observed among the individuals under consideration is summated at each locus along a chromosome. This process is then repeated for each chromosome. Short runs of consecutive SNPs demonstrating no CH occur by chance in unrelated individuals but runs of no CH exceeding a threshold length are indicative of IBD inheritance. To militate against the effect of genotyping errors, single occurrences of CH that directly separate two loci whose combined length exceeds the pre-determined threshold are ignored. CCH analysis contrasts with homozygosity mapping analyses (such as those implemented in PLINK [12] and HomozygosityMapper [13]) which are used to identify loci underlying recessive traits, both in its underlying principle (described above) and because CCH is designed to locate dominant (as opposed to recessive) alleles.

The rapidity of a single genome-wide CH analysis permits repeated analyses to be undertaken testing for IBD inheritance among all *nCk* combinations of subsets of *k* from *n* individuals. The complete independence of each analysis permits extensive parallelisation depending on the available computational resources. CCH is implemented in Python and the source code, along with an example dataset and user instructions are available from http://sourceforge.net/projects/cchsnp/. This program enables analyses to be undertaken, results plotted and includes a feature to permit the generation and processing of segmented files for parallelisation. Scripts to prepare input files from PLINK [12] and high throughput sequencing (.vcf) formats [14] are also provided.

### CH among subsets of unrelated individuals

To determine the distribution of CH among unrelated individuals we used SNP genotypes corresponding to the Illumina HumanCytoSNP 12v2 array (Illumina, CA) of approximately 300,000 SNPs taken from the HapMap Project [15]. Groups of between 8 and 15 unrelated HapMap individuals (identifiers of all individuals utilised are listed in Additional file 1: Table S1) were selected and CCH was used to sequentially analyse all possible subsets of all of these groups. The analysis was repeated 100 times for each group size using different, randomly selected, HapMap individuals. The mean density of this SNP array is approximately 100 SNPs per cM and we exclude all runs of <100 SNPs in length to prevent detecting a false positive signal from regions of the genome with sparse SNP coverage.

### Genotyping and linkage analysis in an example family

All research involving human participants was performed with written informed consent and was approved by the ethics committee of Lefkosa Burhan Nalbantoğlu State Hospital. All participants provided informed consent for their involvement in the research in accordance with the Declaration of Helsinki and for the publication of the study results. Individuals were genotyped on the HumanCytoSNP 12v2 array (Illumina, CA) according to the manufacturers’ instructions. Genotype data have been deposited in the NCBI Gene Expression Omnibus (GEO) [16] and are accessible through GEO Series accession number GSE65312. Standard quality control [17] was undertaken using PLINK [12] to remove SNPs with >5% missingness, <5% minor allele frequency or deviation from Hardy-Weinberg equilibrium (p < 1 × 10^-6^). SNPs demonstrating Mendelian errors were removed. A cohort of 17 unrelated population-matched controls genotyped on the same array were used to prune for LD at r^2^ < 0.2. From these, the SNP with the highest heterozygosity within the family and lowest frequency in the controls within 0.5 cM windows were selected. This yielded 5,344 informative SNPs for linkage. Affected-only parametric linkage analyses was undertaken with SwiftLink [6] using default parameters with a disease allele frequency of 0.0001 and variable phenocopy rates. Input files for SwiftLink were generated using MEGA2 [18]. As SwiftLink utilises an MCMC estimation-based approach it is necessary to repeat each analysis a number of times and take the average LOD score. Each chromosome was thus analysed ten times in parallel.

## Results and discussion

### Marker diversity, linkage disequilibrium, the null distribution and CH threshold

SNPs with low minor allele frequency (MAF) are likely to be identical by state purely by chance, so we sought to determine whether CCH might indicate apparent regions of IBD inheritance because of low marker diversity. However, since it only takes a single instance of CH to break up a haplotype, we hypothesised that CCH analysis would be relatively robust to this effect: very large numbers of consecutive markers showing no CH would be required to mimic the very large (cM-scale) haplotypes inherited within a family. Similarly, local linkage disequilibrium (LD) between nearby markers mean that blocks of alleles tend to be inherited together even in the absence of IBD inheritance from a recent common ancestor. In the Caucasian population, fewer than 10% of SNPs separated by 160 kbp (typically <0.2 cM) show significant evidence of LD, and where the recombination rate (i.e. the number of cM per megabase-pair) is greater, the span of LD tends to be reduced [19]. We therefore hypothesised that such haplotype blocks would also not be large enough to mimic the very large haplotypes inherited within a family.

**Figure 2:**
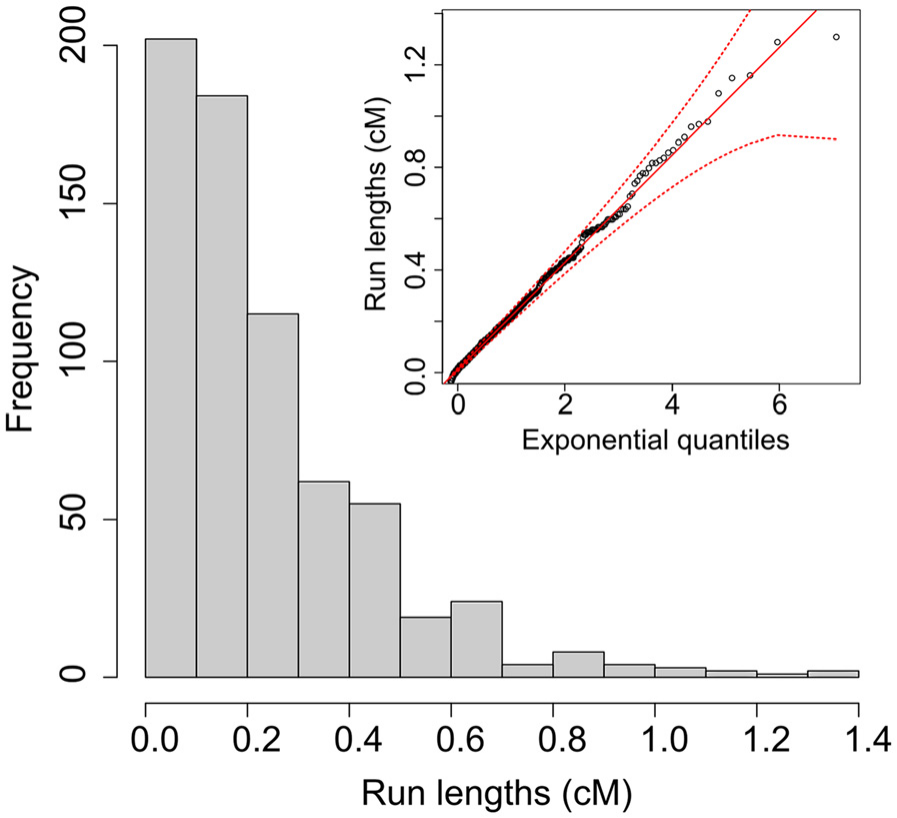
**Histogram showing the distribution of lengths (in cM) of runs of no CH among 11 unrelated people (genotype data taken from HapMap database).** Run lengths were distributed exponentially (inset). A similar distribution was seen for all other groups tested. Dashed lines represent 95% confidence interval.

**Figure 3:**
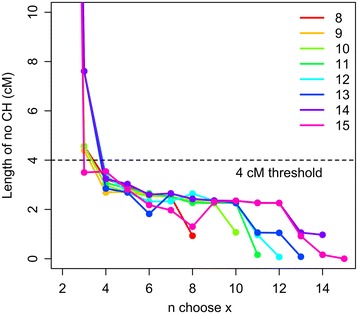
**Length of no CH among all possible subsets of groups of size (n) = 8 to 15 randomly selected individuals from the HapMap database.** 100 replicates of each group size were performed and the maximum run lengths in cM across all replicates of all combinations of possible subsets of every group are plotted (i.e. 6.5 × 10^6^ genome-wide CH analyses). For subsets including 4 or more (unrelated) individuals, no runs extending 4 cM or more were observed.

**Figure 4:**
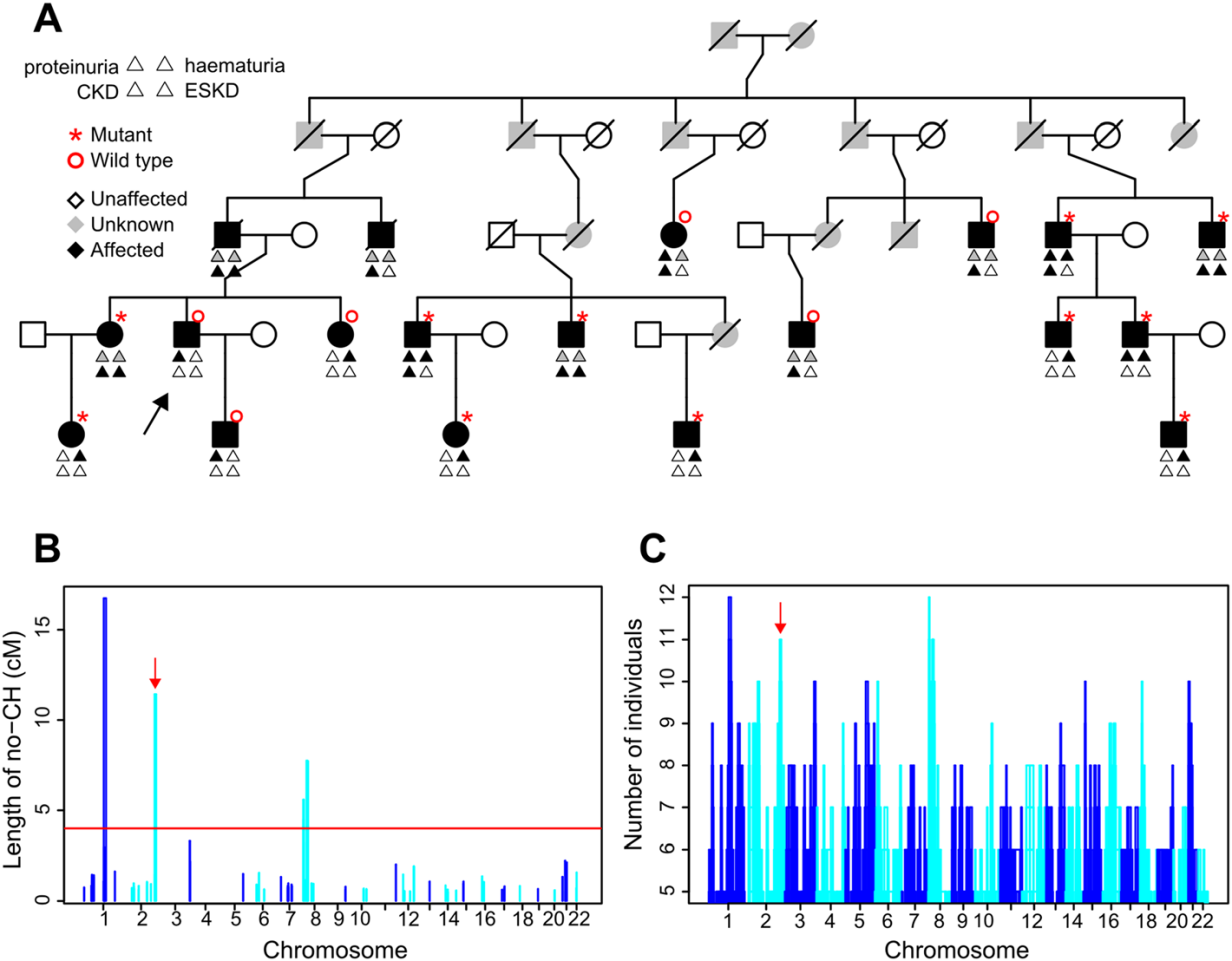
**CCH in a family with kidney disease. A**. Family tree showing affected individuals and connecting relatives only. 29 unaffected 1st degree relatives are omitted. Arrow indicates the proband. CKD: chronic kidney disease, ESKD: end-stage kidney disease. Genotypes for the *COL4A3* p.Gly871Cys mutation are indicated by mutant or wild type respectively, as per the key. **B**. Combinatorial CH analysis showing the 4 loci at which there are runs of no CH ≥4 cM in at least 11 of the 17 clinically affected family members. *COL4A3* is on chromosome 2 (arrow). Colors alternate for clarity. **C**. Genome-wide CCH results showing the maximum number of individuals who are IBD at each locus.

**Figure 5:**
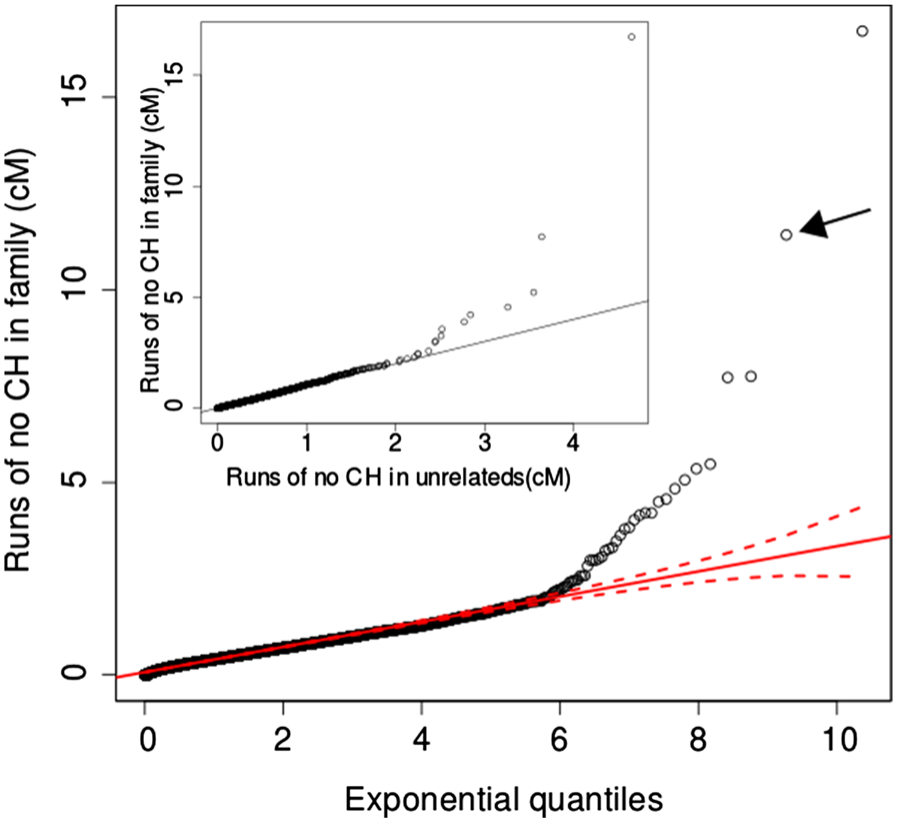
**QQ plot showing lengths of runs of >20 SNPs observed when all combinations of 11 out of 17 affected family members are analysed using CCH and plotted against exponential quantiles.** Arrow indicates the locus spanning the *COL4A3* gene (uncorrected p = 2.5 × 10^-14^ given the underlying exponential distribution). CCH performed with related individuals yields an excess of longer runs compared with the analysis of the same number of unrelated individuals drawn from the HapMap Project (inset).

To test these hypotheses we examined the distributions of no CH run lengths in all possible subsets of between 8 and 15 unrelated HapMap individuals, repeated 100 times using different HapMap individuals. The run lengths (in cM) of no CH observed in these analyses were distributed exponentially (Figure 2) about a mean which varies as a function of the number of individuals being compared. The maximum observed run lengths in cM across all 100 analyses for each group size are plotted in Figure 3. Across all 6.5 × 10^6^ of these genome-wide CH analyses, when subsets comprising 4 or more unrelated individuals (whatever the group size) were included, no runs comprising >100 SNPs and extending >4 cM in length were observed. Since CH cannot occur among a set of individuals at a locus where a haplotype is shared (since at least one allele of each SNP in the shared haplotype must be shared) much longer runs of no CH are expected to occur in regions of the genome where there is IBD inheritance of at least one haplotype from a recent common ancestor. The length of runs arising from IBD inheritance is dependent on the size of the shared haplotype, which decreases as a function of the number of meioses separating the individuals, starting at an expected length of ~34 cM (comprising >3,000 SNPs in the array used in this investigation) for a pair of siblings. When related individuals (Figure 4A) were analysed using CCH, an excess of longer run lengths was observed compared with analysis of the same number of unrelated individuals (Figure 5). These data show that, at least with this 300,000 SNP marker set, regions of low haplotype diversity or strong LD are not large enough to generate a false-positive signal in the absence of recent shared ancestry, and therefore imply that longer runs of no CH are likely to result from IBD inheritance of a haplotype from a recent common ancestor.

### Assessment of statistical significance

The statistical test we use to infer IBD inheritance at a given locus is the likelihood that the observed length of no CH at that locus occurred under the null hypothesis (that is by chance in the absence of recent shared ancestry among the individuals compared).

Using the observed underlying exponential distribution of no CH run lengths (Figure 5) to compute these likelihoods we calculate that for 11 unrelated individuals, the likelihood of observing a run of length >4 cM was <10^-7^ and >7 cM was <10^-12^. For combinatorial CH analyses, these likelihoods should be corrected for the multiple independent analyses performed (using the Bonferroni approach this is equivalent to p < 10^-2^ and p < 10^-7^ for 4 and 7 cM runs, respectively when tens of thousands of combinatorial CH analyses are performed, as in the example pedigree below). Since, in a group of individuals, any IBD locus could harbor the allele responsible for a shared inherited trait, the size of the locus (and hence the p-value for IBD inheritance) does not indicate the likelihood of the allele of interest being located there. Rather, it represents the likelihood that the locus is inherited IBD by all those individuals. This is equivalent in linkage terms to identifying loci which cosegregate perfectly with a trait and therefore exhibit the maximum observed LOD score within a pedigree, with the magnitude of the maximum LOD score depending on the certainty with which haplotypes can be inferred and the family structure. In genome-wide analyses, these IBD loci (whether detected by linkage analysis or CCH) represent the subset of the genome within which a co-segregating allele may lie. When analysing *k* out *n* individuals, the number of independent analyses, *nCk*, is equal to *n*!/(*k*!(*n*-*k*)!). This number rises exponentially as *n* and *n-k* increase, exceeding 10^8^ when *n* > ~30 and (*n*-*k*) > ~10. This places a limit on the size of groups amenable to CCH analysis due both to the computing time required for such a large number of analyses, and also the reduction in statistical power due to multiple independent tests being performed. Nonetheless, CCH analysis remains practical even among large groups, where < ~8 phenocopies are hypothesized.

### Sensitivity and specificity of CCH for detecting IBD inheritance

**Figure 6:**
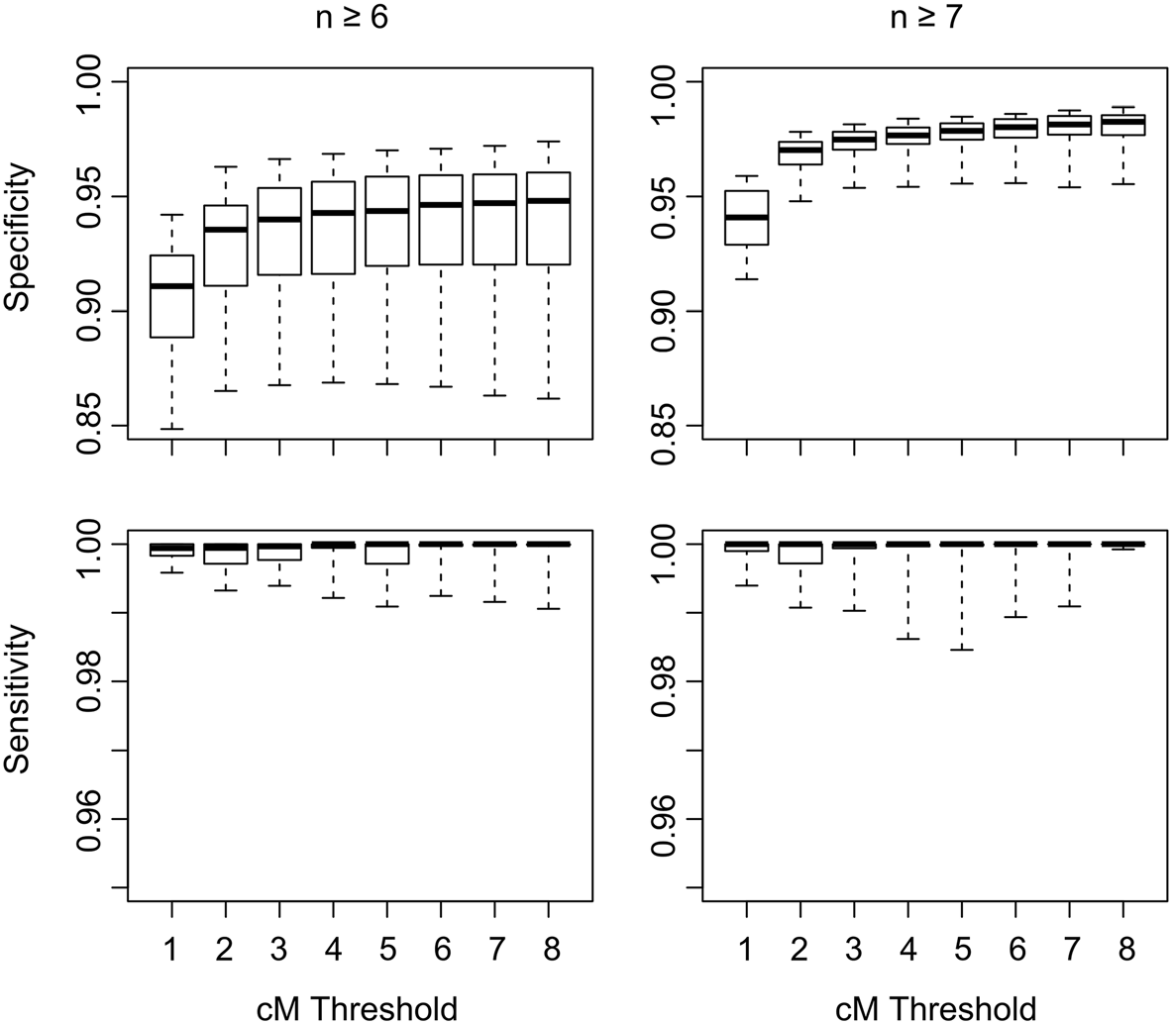
**Sensitivity and specificity of CCH to correctly identify the maximum number of individuals inheriting a haplotype IBD from a recent common ancestor.** Data for ≥6 and ≥7 individuals only are shown.

To assess the sensitivity and specificity of CCH to accurately determine the maximum number of individuals IBD at a locus within a pedigree, Monte Carlo genome-wide gene-dropping simulations using the pedigree structure shown in Figure 4A were performed using Merlin [20] and repeated 100 times. CCH was run on the resulting simulated datasets, with the cM detection threshold varying between 1 and 8 cM (Figure 5). The founder source of each allele in non-founders was determined using founder-specific tags adjacent to each SNP that were removed prior to CCH analysis. CCH was run on each genome-wide simulated dataset testing for loci shared IBD by between 6 and 17 individuals. The maximum number of individuals IBD at each SNP was identified and compared with the real maximum (as determined by the inheritance of the founder-specific tags). Sensitivity was defined as the proportion of SNPs in IBD loci exceeding the cM threshold identified by CCH as being shared by the correct number of individuals. Specificity was the proportion of SNPs identified as IBD by CCH that were genuinely IBD. CCH is highly sensitive, and specificity increased with the cM threshold and the number of individuals included as assessed by comparison with IBD segments from gene-dropping simulations. Using a 4 cM threshold to detect regions inherited IBD in 7 or more individuals genotyped with this ~300,000 SNP array, the median sensitivity was 100% (95% CI: 98.6–100%) and the specificity was 97.7% (95% CI: 95.4–98.4%, Figure 6).

### Double recombinations

When searching for a disease-causing variant within a family, we make the assumption that the variant lies within a shared haplotype that is larger than the detection threshold – double recombination events immediately flanking the variant responsible will therefore prevent its detection by CCH. However, conventional linkage analysis is similarly susceptible to this possibility: linkage is performed either with widely spaced polymorphic markers (typically 300 per genome, mean spacing ~10 cM) or else with a SNP chip using a thinned-out set of perhaps 5–10,000 informative biallelic markers (mean spacing ~0.5 cM) where multiple consecutive markers are needed to reconstruct a haplotype with enough certainty to yield a high-magnitude LOD score. This means that double-recombinations flanking small (i.e. sub-cM) regions will be invisible to haplotype reconstruction algorithms and are therefore missed regardless of the approach used. Because recombinations occur with a median spacing of ~75 cM per meiosis, when dealing with a small number (i.e. <20) meioses in a single family, the probability of recombinations occurring so close together at the disease-causing locus is small. Furthermore, because CCH is robust to the existence of phenocopies, a small number of individuals in whom such a double recombination event has occurred will not prevent detection of the disease-linked haplotype in the rest of the family when CCH is employed. In contrast, the whole locus will be excluded if parametric linkage analysis is performed with a non-negligible phenocopy rate.

### Example: an extended multiplex family with apparently autosomal dominant kidney disease

We investigated a Turkish Cypriot family with apparent autosomal dominant kidney disease (defined as one or more of hematuria, proteinuria or renal impairment) among 19 family members (Figure 4A). No extra-renal manifestations or renal cysts were detected and no individual had undergone a kidney biopsy. Endemic mutations associated with non-cystic kidney disease in the Greek Cypriot population [21,22] were excluded in the proband. The 17 affected individuals from whom DNA was available were genotyped for ~300,000 SNPs.

A genome-wide parametric linkage analysis using a subset of 5,344 informative SNPs took ~105 hours of computing time run in parallel 220 fold (ten repeats of each of the 22 chromosomes) and failed to identify any loci significantly co-segregating with kidney disease (Additional file 1: Figures S1A and S1B). The maximum LOD score was 0.64 at a phenocopy rate of 0.01 and reached 2.25 at a phenocopy rate of 0.05.

Genome-wide CH analysis of all 300,000 SNPs using a standard desktop personal computer took <3 seconds and identified no runs of no CH extending >1.5 cM, consistent with no regions shared by all 17 affected family members. We therefore postulated one or more phenocopies and used CCH to analyse all possible combinations of subsets, of progressively decreasing size, of the 17 clinically affected individuals with a 4 cM threshold. This indicated two loci (of 16.8 cM and 4.5 cM) shared by 12 individuals. These loci contain no genes associated with kidney disease, so further analyses were performed on all 12,376 possible subsets of 11 out of 17 individuals. Together, these analyses took approximately 7 hours to complete serially on a desktop computer and indicated two further loci (of 11.4 and 7.75 cM) shared by 11 individuals (Figure 4B). The 11.4 cM locus included the *COL4A3* gene and molecular testing identified, in all 11 individuals who shared it, a c.2611G > T substitution (chr2:227282487 using GRCh38, predicting p. Gly871Cys) in exon 32 of the *COL4A3* gene. This variant was absent in unaffected members of the family and the six phenocopies, including the index case. Analyses of smaller subsets identified 11 loci shared by ≥10 and 22 loci shared by ≥9 individuals (Figure 4C).

Given that the underlying distribution of no CH run lengths conforms to an exponential distribution (Figures 2 and 5) the likelihood of observing, in the absence of shared inheritance, a run length of >5 cM is <10^-6^ and >7 cM is <5 × 10^-9^. Even with correction for the several thousand combinatorial analyses performed we concluded that such long run lengths were attributable to shared inheritance of a haplotype. To confirm this, haplotypes at loci identified as IBD in >10 individuals were reconstructed using SimWalk2 [23], and co-segregation with the relevant individuals was observed in all cases.

The COL4A3 p.Gly871Cys mutation is associated with kidney disease in the Greek Cypriot population characterised by microscopic hematuria, with proteinuria and progressive renal dysfunction occurring in a proportion of patients in later life [21,24]. Haematuria was present in all eight non-anuric individuals with the mutation and only one of the six phenocopies. We conclude that this mutation explains the kidney disease in these 11 family members. Additional CH analysis demonstrated part of this same haplotype (extending 5.7 cM across the mutation) in an unrelated Greek Cypriot individual with kidney disease who harbored the same mutation, confirming recent shared ancestry in affected individuals from these two communities.

The prevalence of clinical evidence of kidney disease (i.e. proteinuria, hematuria or renal impairment) in the Turkish Cypriot population is not known, however one or more of these abnormalities is detectable in >16% of Australian adults [25]. Considering that clinical data were available for 48 family members, and given a probability of evidence of kidney disease of 16% for each person, under a binomial model that ignores any heritability, the expected number of individuals with evidence of kidney disease is 7.7, which is greater than the six observed phenocopies. Furthermore, the probability of observing >5 affected individuals out of 48 exceeds p = 0.05 when the population prevalence is >5.6%. This implies firstly that additional Mendelian disease need not be invoked in this family to explain the observed number of phenocopies, and secondly that in other families with unexplained kidney disease a similar proportion of phenocopies may be expected.

### Comparison of CCH with parametric linkage analysis

Interrogation of the *COL4A3* locus by linkage at variable phenocopy rates from 0 to 0.45 yielded a maximum LOD score of 1.4 at a phenocopy rate of 0.25 (Additional file 1: Figure S2). Performing a genome-wide analysis at phenocopy rates of 0.25 and 0.35 (corresponding to the actual phenocopy rate at the *COL4A3* locus) identified numerous loci with LOD scores exceeding zero and a smaller number with LOD scores exceeding one (Additional file 1: Figures S1C and S1D). These loci approximately corresponded to those obtained by CCH (Additional file 1: Figure S1E). A flow chart comparing the methodology of CCH with parametric linkage is shown in Additional file 1: Figure S3.

CCH has a number of advantages over tradition linkage approaches: First, CCH directly provides the exact number and identity of individuals sharing each locus (and thus responsible for each linkage signal). Second, CCH only requires genotype data for the affected individuals under examination without a need for data from unaffected connecting relatives. CCH may thus be particularly useful in the context of pedigrees in which affected subjects are distantly related via individuals for whom precise relationship information and DNA is unavailable. Third, pre-processing of data for CCH is minimal only requiring standard quality control procedures with no need to generate an informative marker subset or to provide population specific allele frequencies. Formatting of the data and executing the software is therefore straightforward. Finally, each genome-wide CH analysis of a subset of individuals is rapid (~2.5 seconds) and completely independent the analysis of other subsets, thus permitting significant parallelisation. In the example family under study, to perform linkage analysis using SwiftLink running on a four core processor took an average of ~30 minutes per chromosome. Parallelised 220 fold, each genome-wide linkage took 3.5 hours (including cluster queuing time) with a total running time of ~110 hours. Given that *a priori* the true phenocopy rate is unknown, this would have to be repeated multiple times at different phenocopy rates. By comparison, analysis using CCH of all possible subsets comprising nine or more of the 17 individuals in the family (i.e. without pre-specifying the phenocopy rate) with 200 fold parallelisation per subset size was finished within 30 minutes (including queuing), with a total running time of ~41 hours. The limit on computing time with parallel CCH is simply a product of the number of available cluster nodes.

## Conclusions

CCH can rapidly identify the disease-linked locus in autosomal dominant disease, even in the presence of multiple phenocopies. CCH is also able to detect regions of shared ancestry among ostensibly unrelated individuals using un-phased genotypes. We believe that CCH should be considered in the investigation of inherited diseases where phenocopies are suspected, and may be especially powerful in detecting the location of founder mutations within isolated populations.

## Additional file

Additional file 1:
**Figure S1.** Genome-wide affected-only linkage analysis for the family under study (Figure 4A) undertaken with Swiftlink using an informative LD-pruned marker set at phenocopy rates of 0.01 (A), 0.05 (B), 0.25 (C) and 0.35 (D). For comparison, the CCH results (Figure 4C) are reshown here (E). **Figure S2.** Results of linkage analysis (as per Additional file 1: Figure S1) at the COL4A3 locus at phenocopy rates from 0 to 0.45. **Figure S3.** Flow chart comparing the methodology of CCH with parametric linkage. **Table S1.** Identifiers of 60 HapMap CEU individuals utilised in simulations.
