# Supplementary material for: Combinatorial Conflicting Homozygosity (CCH) analysis enables the rapid identification of shared genomic regions in the presence of multiple phenocopies
Source: BMC Genomics. 2015 Mar 10;16(1):163. doi: 10.1186/s12864-015-1360-4 (PMC4364077; doi:10.1186/s12864-015-1360-4)
Supplement: Additional file 1: — Figure S1. Genome-wide affected-only linkage analysis for the family under study (Figure 4A) undertaken with Swiftlink using an informative LD-pruned marker set at phenocopy rates of 0.01 (A), 0.05 (B), 0.25 (C) and 0.35 (D). For comparison, the CCH results (Figure 4C) are reshown here (E). Figure S2. Results of linkage analysis (as per Additional file 1: Figure S1) at the COL4A3 locus at phenocopy rates from 0 to 0.45. Figure S3. Flow chart comparing the methodology of CCH with parametric linkage. Table S1. Identifiers of 60 HapMap CEU individuals utilised in simulations. [file 12864_2015_1360_MOESM1_ESM.docx]

## Combinatorial Conflicting Homozygosity (CCH) analysis enables the rapid identification of shared genomic regions in the presence of multiple phenocopies

**Adam P. Levine, Thomas M. F. Connor, D. Deren Oygar, Guy H. Neild, Anthony W. Segal, Patrick H. Maxwell and Daniel P. Gale**

Supplementary Figures

**Supplementary Figure 1** Genome-wide affected-only linkage analysis for the family under study (Figure 4A) undertaken with Swiftlink using an informative LD-pruned marker set at phenocopy rates of 0.01 (A), 0.05 (B), 0.25 (C) and 0.35 (D). For comparison, the CCH results (Figure 4C) are reshown here (E).


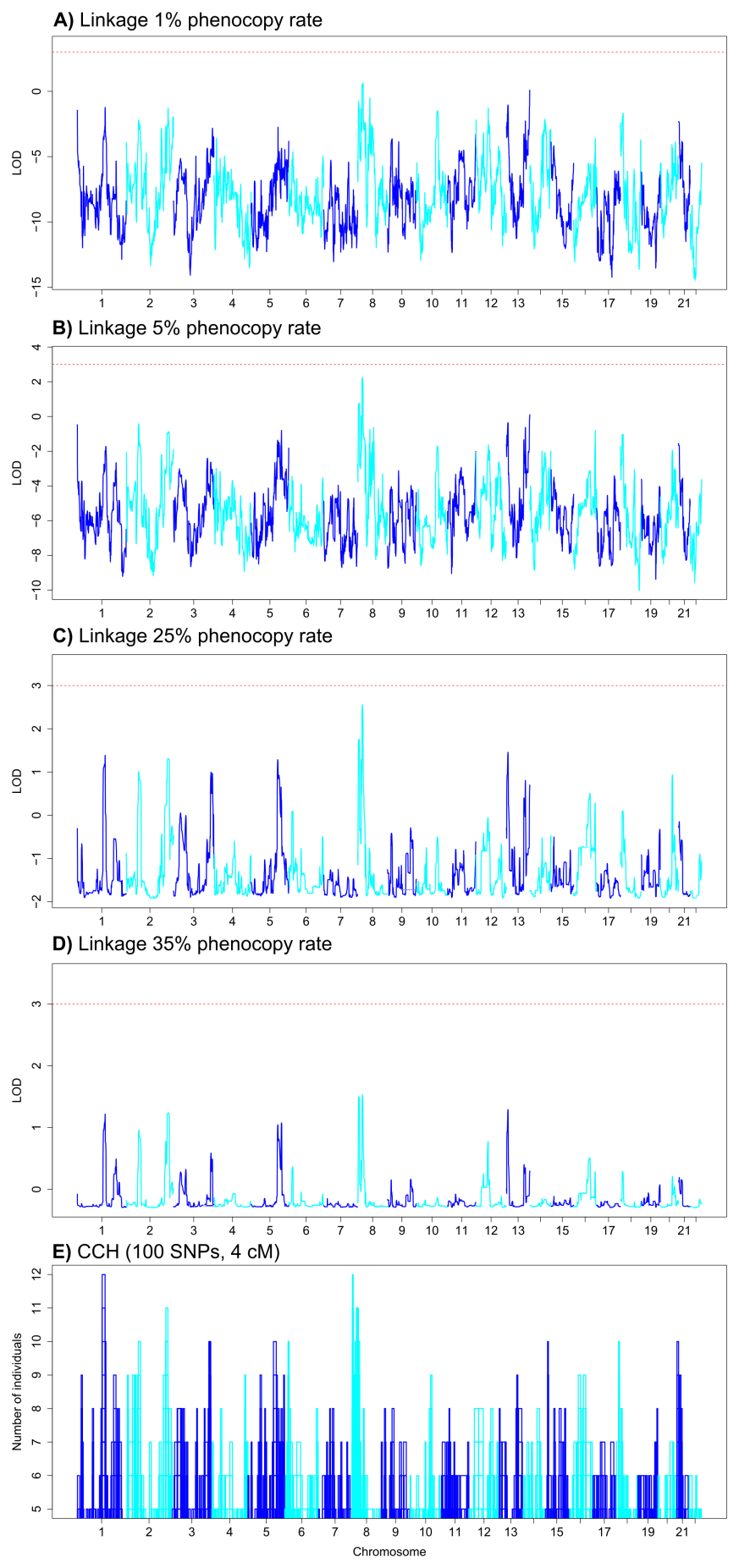


**Supplementary Figure 2** Results of linkage analysis (as per Supplementary Figure 1) at the COL4A3 locus at phenocopy rates from 0 to 0.45.


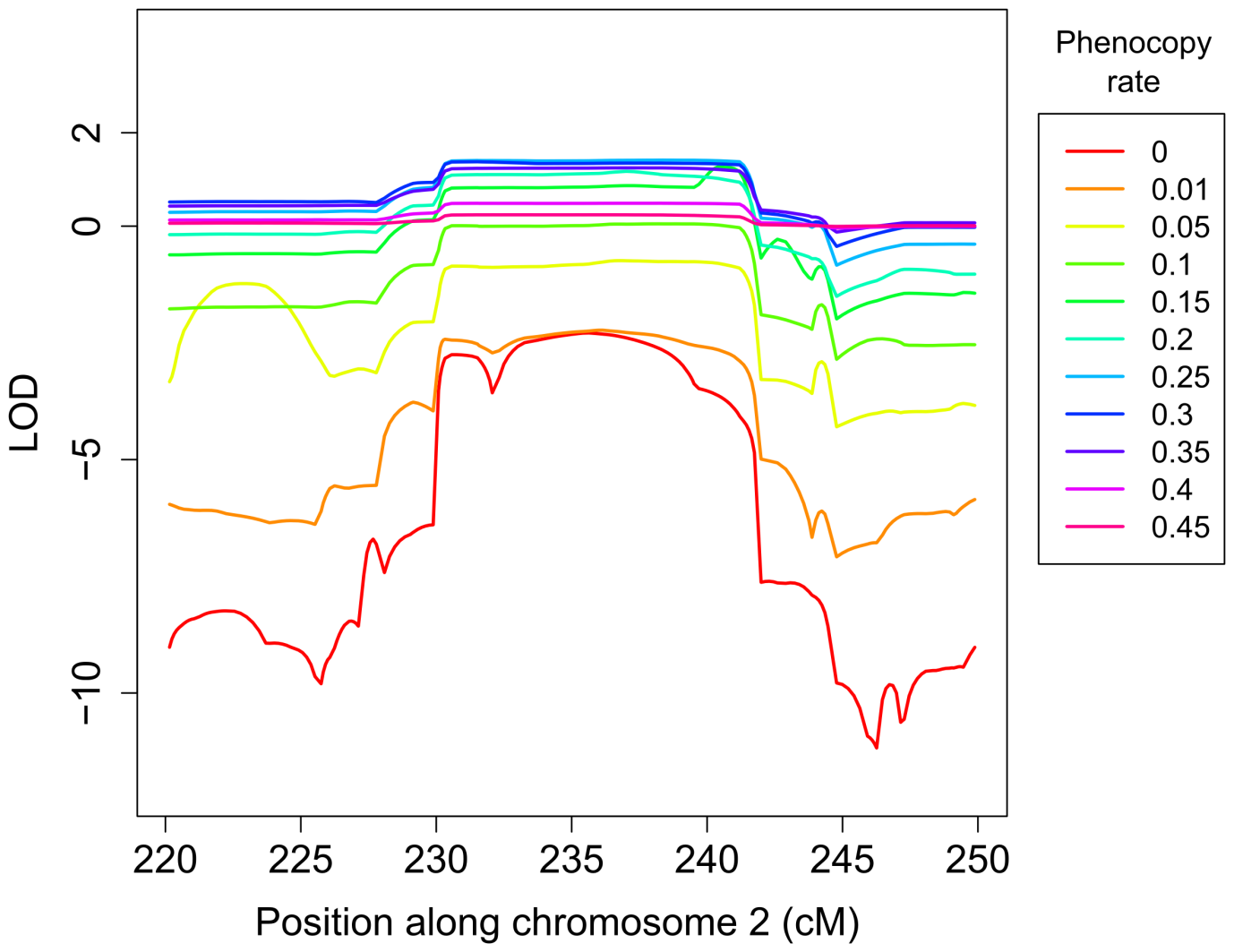


**Supplementary Figure 3** Flow chart comparing the methodology of CCH with parametric linkage.


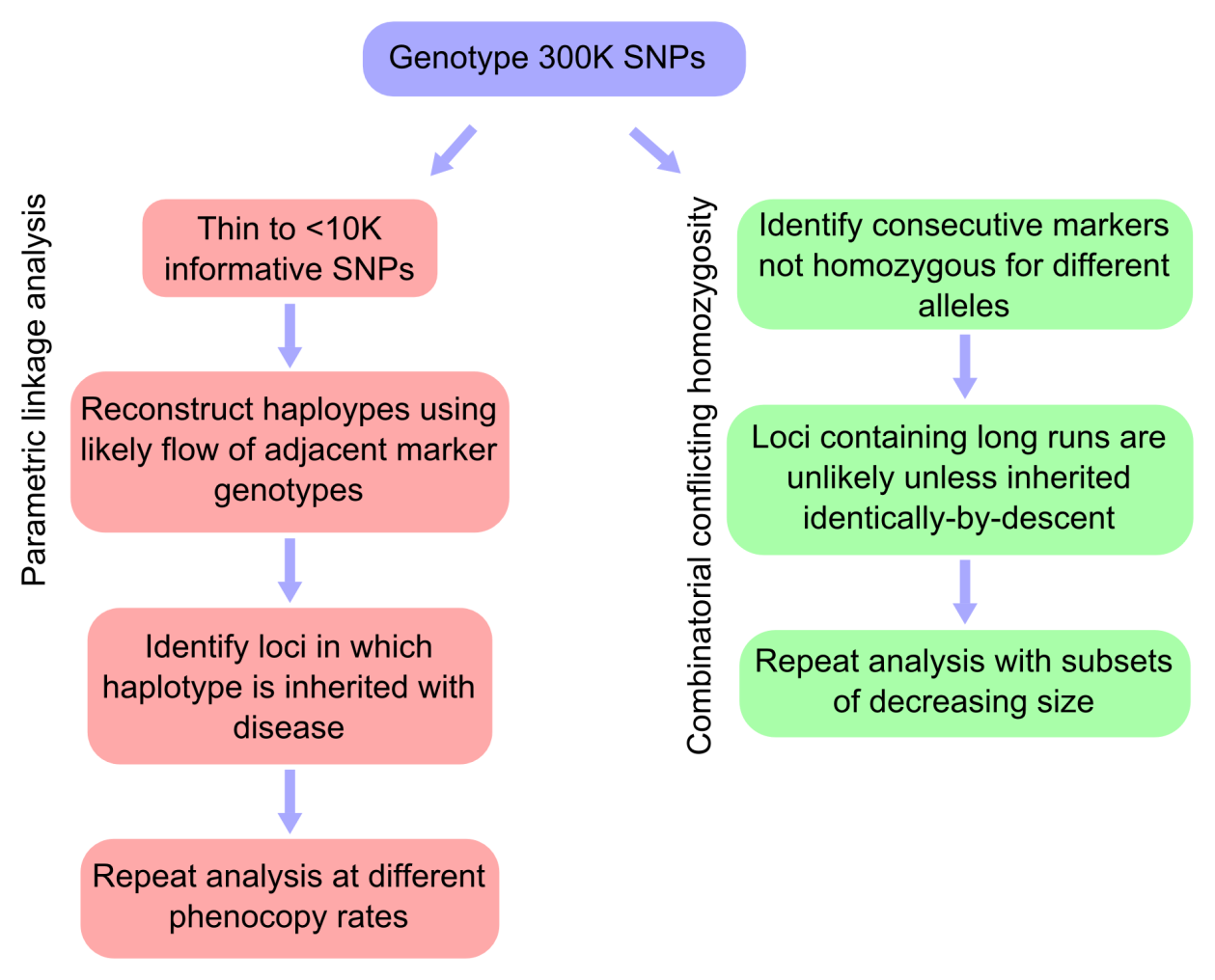


**Supplementary Table 1** Identifiers of 60 HapMap CEU individuals utilised in simulations.

| NA06985 | NA11840 | NA12144 | NA12751 |
| --- | --- | --- | --- |
| NA06993 | NA11881 | NA12145 | NA12760 |
| NA06994 | NA11882 | NA12146 | NA12761 |
| NA07000 | NA11992 | NA12154 | NA12762 |
| NA07022 | NA11993 | NA12155 | NA12763 |
| NA07034 | NA11994 | NA12156 | NA12812 |
| NA07055 | NA11995 | NA12234 | NA12813 |
| NA07056 | NA12003 | NA12236 | NA12814 |
| NA07345 | NA12004 | NA12239 | NA12815 |
| NA07357 | NA12005 | NA12248 | NA12872 |
| NA11829 | NA12006 | NA12249 | NA12873 |
| NA11830 | NA12043 | NA12264 | NA12874 |
| NA11831 | NA12044 | NA12716 | NA12875 |
| NA11832 | NA12056 | NA12717 | NA12891 |
| NA11839 | NA12057 | NA12750 | NA12892 |
